# Assessing and advancing the safety of CRISPR-Cas tools: from DNA to RNA editing

**DOI:** 10.1038/s41467-023-35886-6

**Published:** 2023-01-13

**Authors:** Jianli Tao, Daniel E. Bauer, Roberto Chiarle

**Affiliations:** 1 Department of Pathology, Boston Children’s Hospital and Harvard Medical School, Boston, MA 02115 USA; 2 Division of Hematology/Oncology, Boston Children’s Hospital, Department of Pediatric Oncology, Dana-Farber Cancer Institute, Harvard Stem Cell Institute, Broad Institute, Department of Pediatrics, Harvard Medical School, Boston, MA 02115 USA; 3 Department of Molecular Biotechnology and Health Sciences, University of Torino, Torino, 10126 Italy

**Keywords:** CRISPR-Cas9 genome editing, CRISPR-Cas9 genome editing, Targeted gene repair

## Abstract

CRISPR-Cas gene editing has revolutionized experimental molecular biology over the past decade and holds great promise for the treatment of human genetic diseases. Here we review the development of CRISPR-Cas9/Cas12/Cas13 nucleases, DNA base editors, prime editors, and RNA base editors, focusing on the assessment and improvement of their editing precision and safety, pushing the limit of editing specificity and efficiency. We summarize the capabilities and limitations of each CRISPR tool from DNA editing to RNA editing, and highlight the opportunities for future improvements and applications in basic research, as well as the therapeutic and clinical considerations for their use in patients.

## Abstract (web summary)

CRISPR-Cas tools have shown exceptional promise in genome engineering over the past decade. Here the authors review the development of CRISPR-Cas9/Cas12/Cas13 nucleases, DNA base editors, prime editors, and RNA base editors, as well as their editing precision, off-target effects, and clinical considerations.

## Introduction

Genome editing, which involves the precise and efficient manipulation of DNA sequences within the human genome to alter cell fates and organism traits, has the potential to cure genetic diseases^1,2^. In 2012, the bacterial clustered regularly interspaced short palindromic repeats (CRISPR)-CRISPR-associated protein 9 (Cas9) was engineered for RNA-programmable DNA cutting^3^. Since then, CRISPR-Cas systems have been used to manipulate the genomes of cultured and primary cells^4–7^, animals^8^ and plants^9^, driving major advances in the life sciences and being tested in multiple clinical trials^10–12^. In light of the increasing number of new CRISPR-Cas types^13^, we focus on CRISPR–Cas9/Cas12 in this Review as they are the most widely used genome editors.

CRISPR-Cas DNA editing induces double-strand breaks (DSBs) that are repaired predominantly by non-homologous end-joining pathway (NHEJ) and homology-directed repair (HDR). While NHEJ results in small insertions or deletions (indels) and gene disruption at the target sites, homology-directed repair (HDR) can result in the incorporation of a sequence from the exogenous DNA template at the DSB site, though the efficiency may be limited and is inactive in quiescent cells^14^. In contrast, base editors and prime editors precisely install targeted point mutations without requiring DSBs or donor DNA templates^15^.

CRISPR-associated transposases, Tn6677 and CAST, allow for integration of larger DNA cargos but so far have shown activity only in bacterial cells^16,17^. Briefly, this system uses a combination of several components to install desired cargos (up to 10 kb) at target loci, including the transposase operon (TnsA, TnsB, TnsC, TniQ), donor transposase DNA substrate (LE-cargo-RE), and CRISPR-based DNA-binding and R-loop-generating proteins (cas6, cas7, and cas8-cas5 fusion genes and corresponding CRISPR array for Tn6677, or the cas12k and the Cas12k guide RNA array for CAST). Engineered Cas9-fused integrase/recombinase, which can in theory insert, delete, invert or replace target DNA, have also been reported to modify genomic DNA in mammalian cells, albeit to date with low efficiency and substantial target sequence restrictions^18,19^.

In addition to DNA editing, adenosine deaminases acting on RNA (ADAR) fused to dCas13 have been exploited to achieve precise adenosine-to-inosine editing of target RNA^20^. Unlike genome editing, which is permanent, the effects of RNA editing are transient and reversible. Precise RNA editing may also be achieved by recruiting endogenous ADARs with engineered ADAR-recruiting RNAs, without the need to introduce foreign editing enzymes or exogenous ADARs protein into edited cells^21,22^. Therefore, RNA base editing has therapeutic potential as a complementary approach to genome editing.

In this review, we cover recent advances in the development and application of CRISPR-based genome editing and RNA editing tools, including CRISPR–Cas9/Cas12/Cas13 nucleases, DNA base editors, prime editors, and RNA base editors, focusing on the improvement of their editing efficiency and specificity. We also summarize various NGS-based methods that have been developed to explore unintended genetic alterations and evaluate the safety concerns during gene editing. Finally, we provide perspectives on future directions as well as therapeutic and clinical considerations in this rapidly progressing and exciting field.

## CRISPR–Cas9/Cas12 nucleases

Class 2 CRISPR-Cas systems have been classified into three types, that is type II, type V and type VI, with their corresponding effector proteins Cas9, Cas12, and Cas13, respectively. Among them, most Cas9 and Cas12 variants possess DNA endonuclease activity, while Cas13 variants show preferential RNA-targeting and cleavage activity^15^.

**Fig. 1:**
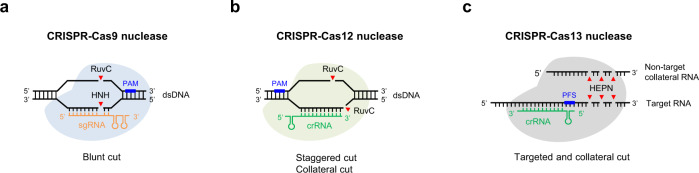
Overview of CRISPR-Cas nucleases. **a**–**c** CRISPR–Cas9 nucleases and CRISPR-Cas12 nucleases are RNA-guided DNA editing tools, whereas CRISPR-Cas13 nucleases have RNA-guided RNA endonuclease activity. Besides targeted cutting, CRISPR-Cas12/13 nucleases also have collateral cutting activity on non-target DNA/RNA templates. *Streptococcus pyogenes* (SpCas9) recognizes a relatively common 3’ NGG PAM, and functions optimally with 20-nt spacers, while Cas12a orthologs generally use 5’ T-rich PAMs. PAM protospacer adjacent motif, PFS protospacer flanking site.

Cas9 nucleases are guided by single guide RNAs (sgRNAs), which were engineered by fusing the CRISPR RNA (crRNA) and trans-activating crRNA (tracrRNA) into a single RNA molecule, predominantly making a blunt-ended double-strand break (DSB) within the protospacer sequence^3^. Mechanistically, following R-loop formation by the Cas9-guide RNA ribonucleoprotein complex, the HNH nuclease domain cleaves the guide RNA-bound target DNA strand, while the RuvC-like nuclease domain cleaves the PAM-containing non-target DNA strand 3 bp upstream of the PAM^23,24^ (Fig. 1a). Cas9 can also generate stagger cutting that resulting in predictable 1-bp insertions^25–27^. In contrast to Cas9, many Cas12 nucleases are naturally guided by a single crRNA. Moreover, Cas12 nucleases possess just a single RuvC-like nuclease domain that mediates cleavage of both target DNA strands, generating staggered DSB cuts within regions of the protospacer that are distal to the PAM sequence^28^ (Fig. 1b).

### Genome-wide off-target editing

Although the target DNA specificity of CRISPR-Cas nuclease is determined by guide RNA, Cas proteins can bind and cleave partially complementary off-target sequences by a range of noncanonical base-pairing interactions within the guide:off-target heteroduplex^29,30^, raising safety concerns for their use in clinical applications and urgent needs to systematically identify these off-target events. Briefly, methods for the genome-wide identification and characterization of potential CRISPR-Cas nuclease off-target sites fall into three general categories: in silico bioinformatic prediction based on sequence homology, in vitro identification of cleavage sites using genomic DNA templates, and in cellulo capturing of off-target editing events^31^ (Table 1). However, no single tool can accurately predict all off-target editing events, especially those at low frequency. In addition, putative off-target sites require further validation by targeted deep sequencing, which is a gold standard assay to verify the presence of nuclease-induced indels/mutations^32^. Besides targeted deep sequencing, other validation methods such as CUT-PCR^33^ and TIDE/TIDER^34^ are greatly limited by their validation scales, as well as limited by the detection sensitivity in the case of sanger sequencing-based TIDE/TIDER^34^. Nevertheless, integration of candidate off-target site libraries by SURRO-seq^35,36^ and an improved dual-target system^37^ allow large-scale validation of CRISPR-Cas nuclease off-targets identified from other methods in a genomic and cellular context, although these methods may still not fully mimic the editor delivery and chromatin state expected in target cells for the gene editing therapy.

**Table 1:** A summary of methods for identifying genome-wide CRISPR-Cas off-target sites

Categories	Detection methods	Key strengths	Key weaknesses
**In silico**	**Bioinformatic prediction based on sequence similarity** Cas-OFFinder, CasOT, E-CRISP, COSMID, CROP-IT, CCTOP, Bowtie2, CNN_std, Elevation, FlashFry, predictCRISPR, Crisflash, Synergizing CRISPR, CRISPRitz, MOFF	High throughput	High false positive
**In vitro**	**Whole-genome sequencing of Cas nuclease-digested DNA/chromatin** Digenome-seq, DIG-seq	High sensitivity	Cost-ineffective
	**DSB end enrichment by DNA circles digestion** CIRCLE-seq, CHANGE-seq	High sensitivity	High skill requirement
	**DSB end enrichment by tagging the cleaved-DNA with Biotin** SITE-seq	High sensitivity	Requires in cellulo validation
**In vitro** **in cellulo**	**Labeling of off-target sites by capturing R-loop mediated ssDNA** CROss-seq	Unbiased	Narrow time-window
**In cellulo**	**Indirect labeling of off-target breaks by repaired products** GUIDE-seq, iGUIDE, GUIDE-tag HTGTS, LAM-HTGTS, PEM-seq, CAST-seq, UDiTaS IDLV capture, ITR-Seq PolyA-seq PEAC-seq, TAPE-seq	High sensitivity High sensitivity; applicable in vivo Unbiased Unbiased High specificity; applicable in vivo	Require efficient dsODNs delivery into cells Require a large number of input DNA Lower-sensitivity; high false positive Requires efficient LINE-1 delivery into cells Requires high prime editing efficiency
	**Indirect labeling of off-target breaks by DNA repair protein MRE11** DISCOVER-seq	Unbiased; applicable in vivo	Narrow time-window
	**Direct labeling of unrepaired break ends** **in situ** BLESS, BLISS	Unbiased	Narrow time-window; high background

Cas-OFFinder is a widely used in silico prediction software for Cas9-induced off-targets^38^. Several web-based prediction algorithms have been developed to rank potential off-targets for particular guide RNAs within the genome, including CasOT, E-CRISP, COSMID, CROP-IT, CCTOP, Bowtie2, CNN_std, Elevation, FlashFry, predictCRISPR, Crisflash, Synergizing CRISPR, CRISPRitz, MOFF, and many others as summarized recently^31,32^ (Table 1). The sequence similarity-based computational algorithms, although powerful and useful, may need more training data and richer biophysical frameworks to fully predict off-target potential. Currently, in silico predictions need to be further supplemented by experimental methods to effectively identify true off-target sites.

**Fig. 2:**
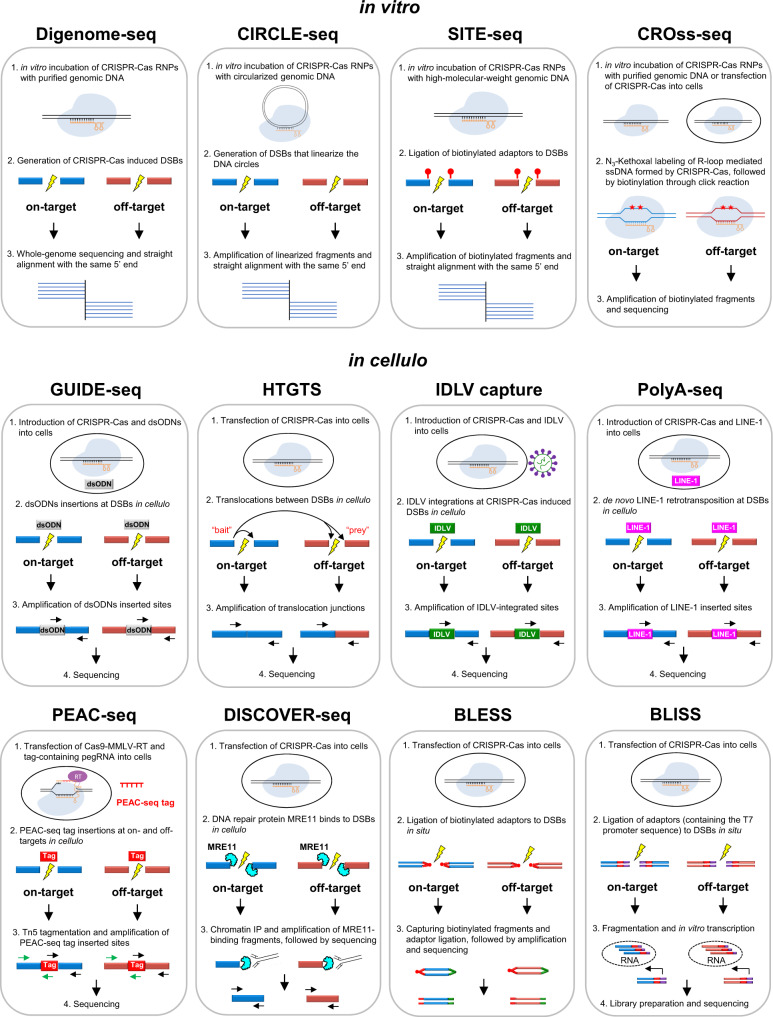
Methods for identifying genome-wide CRISPR-Cas off-target sites. Genome-wide methods for off-target detection can be divided into two groups: cell-free methods (in vitro) and cell-based methods (in cellulo). Cell-free methods include Digenome-seq (digested genome sequencing) and its improved version DIG-seq that use genomic DNA or chromatin as template during in vitro digestion; CIRCLE-seq (circularization for in vitro reporting of cleavage effects by sequencing) and its improved version CHANGE-seq (circularization for high-throughput analysis of nuclease genome-wide effects by sequencing) that use DNA circles as template; and SITE-seq (selective enrichment and identification of tagged genomic DNA ends by sequencing) that tags the DSBs with biotin-labeled adapter for enrichment. CROss-seq (CRISPR Off-targeting ssDNA sequencing) could be used both in vitro and in cellulo, capturing CRISPR-Cas induced R-loops by N_3_-Kethoxal labeling. Cell-based methods include GUIDE-seq (genome-wide, unbiased identification of DSBs evaluated by sequencing) and its improved versions iGUIDE and GUIDE-tag that utilize dsODNs insertions at DSBs in cellulo; HTGTS (high-throughput, genome-wide translocation sequencing), as well as its improved versions LAM-HTGTS and PEM-seq, and similar methods CAST-seq and UDiTaS, can capture translocations between CRISPR-Cas induced on-target (as “bait”) and off-targets (as “preys”); IDLV capture (integrase-deficient lentiviral vector) uses lentiviral vector integrations to identify off-targets, while a similar technique ITR-Seq identifies off-targets by capturing the insertions of specific AAV vector sequences called inverted terminal repeats (ITRs); PolyA-seq detects off-targets by capturing de novo LINE-1 retrotransposon insertions at CRISPR-Cas induced DSBs; PEAC-seq (Prime Editor Assisted off-target Characterization), as well as a similar technique TAPE-seq (TAgmentation of Prime Editor sequencing), tags the on- and off-targets by adopting the prime editor with Cas9 nuclease and a sequence-optimized tag-containing pegRNA; DISCOVER-Seq (discovery of in situ Cas off-targets and verification by sequencing) tracks the precise recruitment of MRE11 at DSBs; and BLESS (direct in situ breaks labeling, enrichment on streptavidin, and next-generation sequencing), as well as its improved version BLISS (breaks labeling in situ and sequencing), directly captures unrepaired DSBs by in situ ligation of biotinylated adapters (for BLESS) or T7 promotor sequence-containing adapters (for BLISS) into off-target breaks.

One class of these experiments-based methods is in vitro identification of the off-target sites using purified genomic DNA and the Cas9 or Cas12 gRNA ribonucleoprotein (RNP) complex. Digenome-seq is a commonly used technique based on in vitro Cas nuclease-digested whole-genome sequencing (WGS)^39^. This in vitro digestion yields sequence reads with the same 5’ ends at cleavage sites that can be computationally identified^39^ (Fig. 2). To mimic the chromatin environment in eukaryotic cells, DIG-seq was developed based on Digenome-seq, using native chromatin for in vitro digestion in contrast to purified genomic DNA in Digenome-seq and thus increasing the validation rate^40^. The WGS-based Digenome-seq and DIG-seq are heavily limited by its cost, since they require high sequencing coverage (∼400-500 million reads) that nearly all of the sequencing reads are background reads (not associated with CRISPR-Cas induced DSBs). CIRCLE-seq utilizes an alternate genomic substrate, DNA circles, for the in vitro genome-wide identification of nuclease off-targets^41^. Mechanistically, genomic DNA is sheared into linear fragments, circularized by intramolecular ligation, and incubated with Cas nuclease during which the circularized DNA molecules containing off-target site are linearized. These linearized fragments are then selectively amplified and sequenced to identify the off-targets^41^ (Fig. 2). CIRCLE-seq greatly reduces the background of endogenous DSBs that occur during biological processes or random DSBs that occur during the genomic DNA purification and processing, as well as reduces the sequencing depth (∼4–5 million reads) which makes it a highly sensitive technique. Furthermore, CIRCLE-seq was improved by CHANGE-seq, a scalable, automatable tagmentation-based method for measuring the genome-wide activity of Cas nuclease in vitro^42^. CIRCLE-seq and CHANGE-seq are limited by the challenges inherit in creating abundant high-quality material, making them much more specialized than Digenome-seq or DIG-seq. Instead of sequencing all the DNA fragments in Digenome-seq, SITE-seq was developed to enrich the nuclease-digested fragments by selectively tagging the cleaved-DNA with biotin-labeled adapter followed by biotin-streptavidin affinity purification and sequencing with a minimal read depth (∼0.62–2.46 million reads)^43^ (Fig. 2). Like other in vitro methods, off-targets found by SITE-seq need further validation from in cellulo methods.

While in vitro cell-free biochemical methods are rapid and conventional, they inevitably capture some pseudo off-target sites compared to the in cellulo methods which require the introduction of Cas nuclease directly into cells to identify genome-wide off-target sites. These unbiased, NGS-based methods fall broadly into four categories: indirect labeling of off-target breaks by repaired products (GUIDE-seq, HTGTS, IDLV capture, PolyA-seq, PEAC-seq), indirect labeling of off-target breaks by DNA repair protein (DISCOVER-seq), direct labeling of unrepaired break ends in situ (BLESS, BLISS), and indirect labeling of off-target sites by capturing R-loop mediated ssDNA (CROss-seq) (Table 1).

GUIDE-seq is the most used method with the help of insertion of a linear double-stranded oligodeoxynucleotides (dsODNs) into CRISPR-Cas off-target sites^44^. Following dsODNs insertion, the specific sequence and adjacent genomic sequences are selectively amplified and sequenced (Fig. 2). Using a larger dsODNs (46 nt versus 34 nt), GUIDE-seq was improved by iGUIDE that allows mispriming artifacts to be distinguished from authentic dsODNs integration sites^45^. Furthermore, GUIDE-tag utilizes tethering between the Cas9 nuclease (SpyCas9-mSA) and the DNA donor (biotin-iGUIDE donor) to increase the off-target capturing rate, as well as UMI incorporation via Tn5 tagmentation to avoid PCR bias^46^. The limitations of GUIDE-seq based methods include the requirement for high transfection efficiency of the dsODNs into cells, and limited use for blunt-ended DSBs. HTGTS was initially developed as a powerful technique that can generate a genome-wide chromosomal translocations map from a fixed DSB “bait” induced by either I-SceI^47^ or CRISPR–Cas9^48^. HTGTS utilizes chromosomal translocations between Cas nuclease-induced on-target DSB (“bait”) and off-target DSBs (“prey”) to capture genome-wide off-target sites (Fig. 2). LAM-HTGTS^49,50^ improves its sensitivity with lower cost, while PEM-seq^51,52^, as well as two similar techniques CAST-seq^53^ and UDiTaS^54^, are further developed to systematically and quantitatively calculate the frequency of different editing outcomes (i.e., indels, off-targets, and chromosomal structural variations) which is especially useful for pre-clinical and clinical evaluations. Although HTGTS-based methods are powerful with high sensitivity, the limitation is Cas nuclease-induced translocations are inherently rare events that require large numbers of input genomes for efficient detection. IDLV capture^55^ and ITR-Seq^56^ use integrase-defective lentiviral vector and adeno-associated viral (AAV) vector to identify off-targets, respectively; while PolyA-seq detects off-targets by capturing de novo LINE-1 retrotransposon insertions at CRISPR-Cas induced DSBs^57^ (Fig. 2). The sensitivity of IDLV capture, ITR-Seq, and PolyA-seq is lower than other in cellulo methods, owing to these methods retain high capability to randomly integrate into genome; in other words, most of the integrations found in IDLV capture, ITR-Seq, and PolyA-seq are genome-wide insertions out of the Cas nuclease-induced DSBs. Similar to GUIDE-seq, PolyA-seq is largely dependent on the efficient delivery of LINE-1 plasmid (∼18 kb) into cells by transfection, as well as the efficient retrotransposition activity in cells. Recently PEAC-seq^58^ and TAPE-seq^59^ were reported to adopt the prime editor to insert a sequence-optimized tag to the on- and off-target editing sites, followed by enriching the tagged regions with primers for high-throughput sequencing (Fig. 2). Since the insertion of tag depends on the sgRNA-sequence, PEAC-seq and TAPE-seq detect off-targets with high specificity, largely excluding labeling the endogenous DSBs. Although these methods heavily rely on the prime editing efficiency, which might vary across different pegRNAs and at different loci, PEAC-seq has been successfully applied for in vivo studies in mouse embryos^58^.

DISCOVER-seq enriches DSBs by immunoprecipitation of MRE11, a protein that specifically binds to DSBs in cells, to detect CRISPR off-targets unbiasedly in vivo^60^ (Fig. 2). Since MRE11 binds to unjoined DSB ends during genome editing, DISCOVER-seq has a narrow time-window to capture Cas nuclease-induced on- and off-targets. Accordingly, the ideal time point for DISCOVER-seq analysis to be carried out should be determined experimentally and may vary between different cell types and methods of CRISPR–Cas9 delivery.

BLESS directly captures unrepaired DSBs genome-wide by in situ ligation of biotinylated adapters into off-target breaks, followed by biotin-streptavidin enrichment and sequencing^61^ (Fig. 2). Since BLESS requires relatively high DNA input leading to a high background, BLISS has been developed to detect DSBs with low-input requirement by linear amplification of dsODNs (containing the T7 promoter sequence) adapters-tagged DSBs via T7-mediated in vitro transcription^62^ (Fig. 2). In contrast to other methods using indirect labeling of off-target breaks by repaired products, BLESS and BLISS can capture DSBs only present at a particular time-window, and can no longer capture DSBs once they have been repaired. Moreover, BLESS and BLISS have relatively high background that may be caused by spurious DSBs introduced during fixation and handling.

As an R-loop structure is formed during CRISPR-mediated RNA-guided site recognition, the opposite ssDNA is exposed which can be labeled by N_3_-Kethoxal^63^ and captured by CROss-seq^64^, representing a new CRISPR off-target identification technique based on ssDNA capturing both in vitro and in cellulo (Fig. 2). The primary limitation is CROss-seq has narrow time-window as it can only capture R-Loop-based CRISPR off-targets, largely excluding the target sites that have been cleaved and/or repaired with indels. Moreover, the correlation between identified R-loop-based off-targets and true off-targets validated on the DNA sequence remains to be determined. Nonetheless, other sensitive methods developed for genomic DSBs detection, including γH2AX ChIP-seq^65^, TC-seq^66^, DSB-Seq^67^, END-seq^68^, DSBCapture^69^, I-BLESS^70^, SAR-seq^71^, and INDUCE-seq^72^, could also be modified and used for the detection of CRISPR off-target sites.

Currently, there is no single method optimized for all contexts in which the off-target activity of CRISPR-Cas nuclease needs to be assessed. Thus, the best strategy may be the use of multiple approaches to assess and validate the off-target effects. The choice may be driven by considering the strengths and weaknesses of each approach as summarized in Table 1, together with different experimental settings or clinical demands. For example, if the cells can be efficiently transfected, GUIDE-seq and PolyA-seq provide effective, sensitive, and rather simple methods for off-target detection. IDLV capture or ITR-Seq could be a good alternative in cases where viral transduction is more practical and efficient than dsODNs or LINE-1 plasmid transfection. In vitro methods could be useful when transfection and/or transduction of cells is inefficient, although the identified off-targets would eventually need to be further validated in cellular context. DIG-Seq is an improved in vitro method that maintains the chromatin architecture, and consequently has a higher validation rate compared to Digenome-seq, CIRCLE-seq and SITE-Seq^40^. HTGTS-based methods, as well as other DSBs-capturing methods, are powerful in identifying CRISPR-Cas off-target activity that results in the formation of DSBs. In contrast, these methods are difficult to capture mutations-mediated off-target events such as some off-target events induced by base editors.

### Genomic rearrangements

Though genome editing with CRISPR–Cas9 holds great promise for the treatment of human genetic diseases, it results in a mix of intended and unintended genetic alterations. More rarely, DSBs induced by CRISPR–Cas9 can lead to larger genomic rearrangements including large chromosomal deletions, inversions or translocations^25,49,73–81^ or even more catastrophic events such as chromothripsis^82^, aneuploidy^83,84^, chromosome loss^85–87^ and p53 activation that can enrich oncogenic cells^88–90^. Additional outcomes of the CRISPR–Cas9 editing include integrations of exogenous sequences including lentivirus^55,57,91^, adeno-associated virus (AAV)^56,92^, plasmids^25,57,78,80,93,94^ and small DNA fragments^44,95^. Recently, we reported that LINE-1 retrotransposons can occur at CRISPR–Cas9 editing sites, in a reverse transcriptase (RT) activity-dependent and endonuclease (EN) consensus motif-independent manner^57^. These events exploit the availability of frequent DSBs generated by CRISPR–Cas9 nuclease to favor the insertion of exogenous DNA fragments using microhomology-mediated end joining (MMEJ)^25,57,92,96^. Although chromosomal rearrangements are likely rare events, the concern is that even a very small number of CRISPR–Cas9-edited cells harboring these detrimental events may clonally expand in vivo^97^ and cause diseases.

### Methods for reducing off-target effects and genomic rearrangements

The way to reduce off-target effects is to optimize the different components of the CRISPR-Cas systems. The selection of sgRNAs that yield high editing efficiency balanced with low off-target activity is likely one preferred strategy for increased safety, especially for clinical applications. In addition, several modifications have been developed for reducing CRISPR nuclease-induced off-target effects and genomic rearrangements while retaining robust on-target activity, including sgRNA modifications, Cas9 protein optimizations, Cas9 system modifications, and delivery improvements.

Truncated sgRNAs (tru-gRNAs) (with only 17 or 18 nucleotides)^98^, chemically modified sgRNAs (2’-O-methyl-3’-phosphonoacetate, or “MP” modification)^99^, light-induced degradable sgRNA^100^, and incorporated bridged nucleic acids (2’,4’-BNANC[N-Me]) or locked nucleic acids (LNA) at specific locations in crRNAs^101^ have been shown to broadly reduce off-target activity of CRISPR–Cas9 by several orders of magnitude. Interestingly, since short gRNAs (less than 16 nt in length) effectively recruit Cas9 to complementary sites in the genome but do not permit Cas9 nuclease activity, CRISPR GUARD was developed as a method for protecting off-targets sites by co-delivery of short gRNAs to compete with the on-target sgRNA^102^, similar to co-administration of dead-RNAs (dRNAs)^103^.

Besides sgRNA modifications, high-fidelity SpCas9 proteins are generated and further optimized. eSpCas9 (K810A or K848A, K1003A, and R1060A)^104^, FeCas9 (K848A, K1003A, R1060A, and D1135E)^51^, and SpCas9-HF1 (N497A, R661A, Q695A, and Q926A)^105^ attenuate contacts with the phosphodiester backbone to be trapped in an inactive state when bound to mismatched targets, while HypaCas9 (N692A, M694A, Q695A, and H698A)^106^ increase the stringency of the allosteric regulation of HNH domain activity with high genome-wide specificity. HiFi Cas9 (R691A) demonstrates superior on-target editing with RNP delivery compared to eSpCas9 and SpCas9-HF1, with decreased off-target activity^107^. In addition, evoCas9 (M495V, Y515N, K526E, and R661Q)^108^, Sniper Cas9 (F539S, M763I, and K890N)^109^, xCas9 (A262T, R324L, S409I, E480K, E543D,M694I, and E1219V)^110^, Cas9_R63A/Q768A^111^ and SuperFi-Cas9^30^ were reported to have lower off-target activity.

A SpCas9 nickase cleaves only one DNA strand by mutating one of the nuclease domains (D10A or H840A). Double nicking by paired Cas9 nickases showed greatly enhanced genome editing specificity^49,112–115^. The Wolfe laboratory developed a Cas9-pDBD (programmable DNA-binding domain) system that restricts its ability to engage its target site, increasing specificity up to 160-fold at off-target sites^116^. Fusing a minimal motif to spCas9, MiCas9 increases large-size gene knock-in rates and reduces undesirable on-target and off-target indels by the enrichment of RAD51 at target sites^117^. Small indels and large deletion frequency are shown to be decreased in cells deficient for the central resection gene *Nbn* and the microhomology-mediated end-joining gene *Polq*^81^. The Hu laboratory reported the generation of a high level of translocations is dependent on repeated cleavage at the Cas9-targeting sites^118^. Thus, Cas9TX is generated by fusing Cas9 with optimized TREX2, an exo-endonuclease that prevents perfect DNA repair and thereby avoids repeated cleavage, eliminating chromosomal translocations during genome editing both in cell models^118^ and a mouse model of age-related macular degeneration^119^. More recently, they showed that compared to Cas9 and Cas12a nucleases, Cas12f nucleases reduce the levels of off-target effects, chromosomal translocations, large deletions, and plasmid insertions^80^.

CRISPR-Cas can be delivered into cells either as plasmid DNA or mRNA encoding their expression, or directly as proteins or ribonucleoproteins (RNPs)^2^. RNPs have been shown to reduce off-target effects as they act on-target DNA immediately after transfection and are rapidly degraded by proteases and ribonucleases in cells^120–122^. For example, exosome-mediated delivery of Cas9 RNPs have been shown for tissue-specific gene therapy of liver diseases^123^. Moreover, considering the numerous plasmid DNA or viral DNA integrations at editing sites, RNPs could be a safer delivery method for CRISPR-Cas tools. Though the endogenous LINE-1 retrotransposons may still be a potential threat during RNPs-mediated CRISPR-Cas editing, base editors or prime editors could be used as a safer alternative consistent with their reduced DSBs^57^.

## DNA base editors

**Fig. 3:**
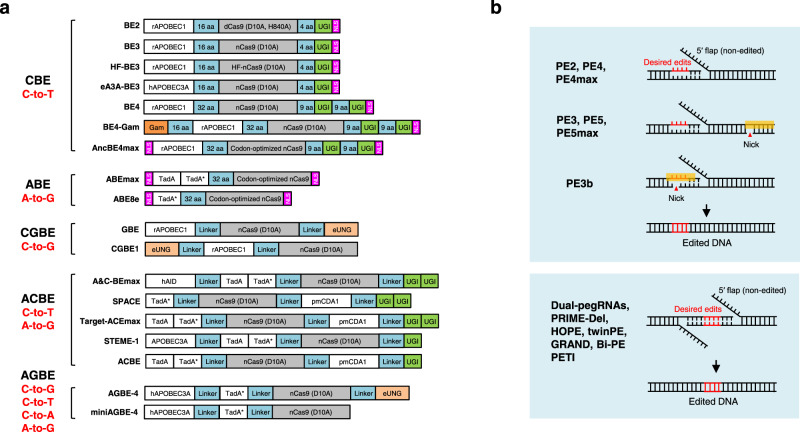
Overview of DNA base editors and prime editors. **a** Diagram of the DNA base editors. BE2, BE3, HF-BE3, eA3A-BE3, BE4, BE4-Gam, and AncBE4max are developed as cytosine base editors (CBEs), while ABEmax and ABE8e are adenine base editors (ABEs). GBE and CGBE1 belongs to C-to-G base editors (CGBEs). A&C-BEmax, SPACE, Target-ACEmax, STEME-1 and ACBE are generated as dual-deaminase base editors (ACBEs) by fusing CBE with ABE. When fused a CGBE with ABE, AGBEs are developed as represented by AGBE-4 and miniAGBE-4. **b** Diagram of the Prime editors. While nCas9 (H840A) and pegRNA are required for all prime editing strategies, PE3/PE5/PEmax contains a nicking sgRNA to increase the editing efficiency. PE3b contains a sgRNA with spacer that match the edited strand to minimize the presence of concurrent nicks. Besides one pegRNA-mediated conventional PEs, two pegRNAs strategy is used in dual-pegRNAs, PRIME-Del, HOPE, twinPE, GRAND, Bi-PE and PETI to further increase the prime editing efficiency, enable longer edits and introduction of recombination sites.

Many genome editing applications require the introduction of precise point mutations, small insertions, or deletions to install or correct the pathogenic mutations in the human genome^15^. Two classes of DNA base editors, cytosine base editors (CBEs) and adenine base editors (ABEs), have been developed to precisely install targeted point mutations without requiring DSBs or donor DNA templates^14^. In CBEs, the cytidine deaminase APOBEC/AID is fused with nickase Cas9 for targeting via sgRNA and with UGI to increase the accuracy and efficacy of base editing, converting C•G base pairs to T•A base pairs^124,125^. In ABEs, a laboratory-evolved TadA deaminase is fused with Cas9 nickase to convert A•T base pairs to G•C base pairs^126^. Similarly, C-to-G base editors (CGBEs) (such as GBE^127^, CGBE1^128^ and other CGBEs^129–131^) consist of a Cas9 nickase, a cytidine deaminase and a uracil-DNA glycosylase (Ung), converting C•G base pairs to G•C base pairs, expanding the editing scope of base editors (Fig. 3a).

Briefly, in contrast to dCas9-fused BE2, BE3 uses Cas9 nickase (D10A) to specifically nick the non-edited strand to increase the editing efficiency^124,132^. Extending linker length and appending a second copy of UGI, BE4 performs base editing with higher efficiency and greatly improved product purity compared to BE3^133^. Fusing BE4 with Gam, a bacteriophage Mu protein that binds DSBs, BE4-Gam greatly reduces indel formation during base editing^133^. Optimization of codon usage and nuclear location sequences (NLS) resulted in the most advanced and efficient AncBE4max or ABEmax^134^, though this increased activity may also increase the off-target effects. ABEmax was optimized from ABE7.10 and consists of a wild-type TadA monomer and an evolved TadA monomer, while the wild-type TadA monomer has been shown not required for ABE activity^135,136^. Thus, ABE8e was developed with only one phage-assisted further evolved TadA monomer, showing the highest efficiency^136^ (Fig. 3a).

Furthermore, dual-deaminase base editors (ACBEs) (including A&C-BEmax^137^, SPACE^138^, Target-ACEmax^139^, STEMEs^140^, and ACBE^141^) have been created by fusing cytidine deaminase and adenine deaminase together with Cas9 nickase to concurrently achieve C-to-T and A-to-G conversions at the same target site. More recently, the Lai laboratory fused a CGBE with ABE to develop a new type of dual-deaminase-mediated base editing system, called AGBE system, that can simultaneously introduce 4 types of base conversions (C-to-G, C-to-T, C-to-A, and A-to-G) as well as indels and thus can be used for saturated mutagenesis^142^ (Fig. 3a).

### Off-target DNA editing and RNA editing of DNA base editors

Though DNA base editors may be more precise tools compared with CRISPR-Cas nuclease^57^, sensitive and unbiased detection of their off-target effects remain important for BE application to human therapeutics. There are two types of off-target activities of CBEs and ABEs: Cas-dependent off-target events caused by the similarity of the sequences within a given mismatch tolerance and Cas-independent off-target events resulting from the nontargeted binding of deaminase to the off-target sites. With the help of GOTI (genome-wide off-target analysis by two-cell embryo injection), the Yang laboratory showed that in mouse embryos, CBEs generate genome-wide substantial off-target single nucleotide variants, which are enriched in the transcribed regions of the genome^143^, similar to another excellent work in rice^144^. These findings were further confirmed and expanded by Digenome-seq^145,146^, EndoV-seq^147^, Orthogonal R-loop assay^148,149^, and CROss-seq^64^, showing ABEs could also generate genome-wide off-targets at a much lower frequency.

Besides DNA off-targeting, transcriptome-wide RNA-seq was also used to detect off-targets at the RNA level. Three independent groups demonstrated that both CBEs and ABEs can cause substantial (tens of thousands of) off-target RNA SNVs in human cells^135,150–152^, consistent with the RNA editing ability of deaminase APOBEC1 or TadA.

### Out-of-protospacer editing and target-strand editing of CBE

CBEs catalyze C-to-dU conversions and finally result in C-to-T transitions^124^. Deoxyuridine (dU) capturing has been well established by NGS-based high-throughput methods such as Excision-seq^153^, dU-seq^154^, UPD-Seq^155^, U-DNA-Seq^156^ and AI-seq^157^. By capturing the base editing intermediate dU, a more advanced technique Detect-seq^158^ is able to trace both the on-target and off-target editing events of CBE, as recently validated by a similar technique called Ucaps-seq^159^. Mechanistically, Detect-seq uses an in vitro-reconstituted base-excision repair (BER) reaction to achieve specific labeling of dU, during which the normal dTTP or dCTP are replaced by biotin-dUTP or 5-formyl-deoxycytidine triphosphate (5fdCTP) to allow biotin pulldown of the dU-containing DNA or tandem C-to-T transitions (used to amplify the signal), respectively^158^. In addition to Cas9-dependent and Cas9-independent off-targeting, Detect-seq discovered CBE edits outside the protospacer sequence (out-of-protospacer editing) and on the non-edited strand (target-strand editing), expanding the knowledge that CBEs could induce proximal off-target mutations^158^.

DddA-derived mitochondrial base editors (DdCBEs), which are fusions of split DddA halves and transcription activator-like effector (TALE) array proteins, enable targeted C-to-T conversions specifically in mitochondrial DNA^160^. Intriguingly, using the same technique, Detect-seq found that DdCBEs could induce extensive off-target editing in the nuclear genome in a TALE array sequence (TAS)-dependent or TAS-independent manner^161^. Importantly, the authors engineered DdCBEs in three different ways to alleviate such nuclear off-target effects.

### Methods for reducing off-target effects of DNA base editing

Strategies to reduce off-target effects of DNA base editors are similar to CRISPR-Cas nuclease, including modified guide RNAs, engineered Cas9, delivery improvements, and engineered high-fidelity cytidine or adenosine deaminase.

High-fidelity Cas9 variants, such as eSpCas9, SpCas9-HF, HypaCas9, and SuperFi-Cas9 in the form of nickase, were incorporated into BE architecture to decrease Cas-dependent off-target effects^132,162,163^. A Cas-embedding strategy, which introduces deaminase (APOBEC1 or TadA) into the middle of Cas9n at tolerant sites instead of the N-terminus of nCas9, significantly reduces gRNA-independent off-target editing by DNA base editors^164^. In contrast to plasmid DNA/viral delivery, delivery of the BE systems via RNP complex or mRNA/sgRNA or lipid nanoparticles (LNPs) result in more transient editing activity and hence improved specificity with fewer off-target effects^132,145^.

By engineering cytidine or adenosine deaminase, several high-fidelity BE variants have been reported to minimize the DNA and RNA off-targeting^165^. In CBEs, these variants include SECURE-BE3^150^, eA3A-BE3^152,166^, hA3G-CBE^167^, AALN-BE4^148^, YE1-BE4^148^, transformer BE (tBE)^168^, AID-2S/AID-3S^169^, and other next-generation CBEs^170,171^. In ABEs, SECURE-ABE^135^, ABE7.10-F148A^152^, ABEmaxAW or ABEmaxQW^151^, ABE8e-V106W^136^, ABE-del153^172^ and ABE9 (N108Q/L145T)^173^ were created to reduce RNA off-targeting and narrow the editing window. Of note, many of these mutations may reduce the deaminase activity. Thus, increased specificity could be a tradeoff for reduced efficiency in selective therapeutic contexts.

Since ABEs show lower DNA and RNA off-target activity, smaller size and higher on-target editing efficiency than CBEs, two groups re-engineered ABEs for efficient and specific CRISPR-based cytosine base editing^174,175^. Evolved TadA cytidine deaminases contain mutations at DNA-binding residues that alter enzyme selectivity to strongly favor deoxycytidine over deoxyadenosine deamination, representing the first unnatural cytosine deaminases besides the natural AID/APOBEC protein family. Briefly, Td-CGBE performs highly efficient and precise C-to-G editing^175^, while Td-CBEs^175^ and TadCBEs^174^ generate precise C-to-T base conversions with the fusion of UGI and additional mutations. Moreover, TadA dual-base editor (TadDE) was shown to perform equally efficient cytosine and adenine base editing concurrently (C-to-T and A-to-G)^174^, providing a highly efficient and small size alternative besides the previously reported dual editors that fuse both cytidine and adenosine deaminases (ACBEs)^137–141^, as well as enabling saturation mutagenesis and screening.

## Prime editors

Prime editors (PE) enable precise genome editing, including targeted small insertions, small deletions, and all 12 possible types of point mutations and their combinations without requiring DSBs or donor DNA templates. PEs use Cas9 nickase (H840A) fused to an engineered Moloney murine leukemia virus (M-MLV) reverse transcriptase (RT) that is programmed with desired edit-containing pegRNA. The pegRNA contains the sgRNA, a primer binding site (PBS) and a reverse RNA template (RTT) for a reverse transcriptase to generate the desired edit at the target site^176–178^.

Specifically, PE2 nicks the target site to expose a 3′-OH that could be used to prime the reverse transcription of pegRNA into the edited strand, while PE3 makes a second nick on the non-edited strand to induce its replacement and hence increase the editing efficiency. PE3b was then developed as an improved strategy that nicks the non-edited strand only after edited-strand flap resolution to minimize the presence of concurrent nicks and potential DSB in PE3^176^ (Fig. 3b). Furthermore, PE4 and PE5 were developed based on PE2 and PE3 systems respectively, enhancing editing efficiency through co-expression of dominant-negative MLH1 (MLH1dn) to inhibit DNA mismatch repair (MMR)^179^, consistent with another paper^180^. Optimization of the prime editor proteins resulted in the most advanced PE4max and PE5max^179^, while the editing efficiency can be further increased by synonymous mutations in pegRNA^181^ or engineered pegRNAs to stabilize the RNA structure^179,181–185^. Other optimizations of prime editing include adding the Rad51 DNA-binding domain^186^, optimized NLS composition^187^, co-selection with puromycin^188^, fluorescent reporter^189,190^ or cellular resistance^191^, peptide fusion^192^, transient inhibition of p53^193^, split prime editor with untethered reverse transcriptase^185,194^ or split-intein prime editor^187^, removing the ribonuclease H domain of M-MLV RT and incorporation of a viral nucleocapsid protein with nucleic acid chaperone activity^195^. Moreover, Cas9 nuclease-based prime editors have been shown to improve PE efficiency, but it comes at the expense of product purity^188,196,197^.

Instead of single-pegRNA-mediated PE system, several groups independently claimed that two pegRNAs encoding the same edits in both sense and antisense DNA strands enable programmable replacement or large deletions with high editing efficiency (Fig. 3b). This excellent work includes dual-pegRNAs developed in plants^198^, PRIME-Del^199^, HOPE^200^, twin prime editing (twinPE)^201^, GRAND editing^202^, Bi-PE^203^, and PETI^204^, as well as a similar technique PEDAR that fuses Cas9 nuclease to reverse transcriptase (PE-Cas9) and combining it with two pegRNAs^205^. Besides highly efficient replacement and large deletions, twinPE enabled precise large insertions (gene-sized length, >5000 bp) or large inversions (40 kb) in human cells when combined with a site-specific serine recombinase, expanding the capabilities of gene editing for the correction of large or complex human pathogenic alleles^201^, similar with PASTE which uses a CRISPR–Cas9 nickase fused to both a reverse transcriptase and serine integrase for targeted genomic recruitment and integration of desired payloads (up to 36 kb)^206^.

### Safety evaluation and improvements of prime editors

Owing to the potential DSBs created by two nicks in PE3, there is a certain level of genomic fragments, plasmid and LINE-1 insertions observed at on-target sites edited by PE3, though at much lower frequency than CRISPR–Cas9 nuclease; while PE2 or PE3b editing has rare insertions consistent with their reduced DSB formation^57,176^. Likewise, using two pegRNAs could also create DSBs; thus, their off-target and unintended on-target effects need to be carefully evaluated. Additionally, several studies have shown that reverse transcription of 3’-extended pegRNAs (RTT-PBS sequence) can proceed into the tracrRNA scaffold, resulting in massive scaffold sequence insertions at the prime editing site with various insertion lengths^57,176,207^. Therefore, splitting the pegRNA into sgRNA and a separate RTT-PBS sequence (either in a linear or circular form) not only prevent 3’-extended pegRNAs degradation by nucleases^185^, but also may be used to reduce the pegRNA scaffold insertions.

Meanwhile, the Cas-dependent and Cas-independent off-target effects of prime editors have been investigated. Nickase-based Digenome-seq (nDigenome-seq)^208^, CROss-seq^64^, and TAPE-seq^59^ were used to screen for genome-wide Cas-dependent off-target sites of PE systems, demonstrating that PEs provide highly specific genome editing with very limited off-targets which could be further improved by high-fidelity Cas9 variants^208^. On the other hand, three recent studies showed PE3 does not induce significant Cas-independent off-target mutations/rearrangements in plants^209^ or human cells^210,211^ both in genomic and transcriptomic level, suggesting high editing specificity of its reverse transcriptase moiety.

## RNA editing

The class 2 type VI systems with the single effector protein Cas13 are the only CRISPR-Cas systems known to exclusively target RNA, showing promise as tools for knockdown, detection and RNA editing^212^. As an RNA-guided RNA endonuclease (RNase), Cas13 consists of two HEPN domains that together form a composite RNase active center responsible for catalyzing RNA cleavage after target RNA binding^213,214^. Cas13a, Cas13b, Cas13d-mediated RNA degradation and gene knockdown exhibit high efficiency and specificity relative to RNA interference, enabling potential therapeutic treatments including nervous diseases and pathogenic viruses^215–217^.

Interestingly, activated Cas13-crRNA complexes cleave both target RNA (cis-cleavage) and non-target collateral RNAs (trans-cleavage)^218,219^ (Fig. 1c), making it possible for sensitive nucleic acid detection and diagnostics. For example, SHERLOCK (specific high-sensitivity enzymatic reporter unlocking) has been developed based on Cas13 for nucleic acid detection^220,221^, as well as adapted for detection of SARS-CoV-2 with an updated version called STOP (SHERLOCK testing in one pot)^222^. On the other hand, collateral degradation of bystander RNAs by CRISPR-Cas13 system has limited its applications in vivo. Hence high-fidelity Cas13 variants with minimal collateral effects have been developed, which could further elevate their utility for targeted degradation of RNAs in basic research and therapeutic applications^223^.

### RNA base editors

Similar to DNA base editors, catalytically inactivated Cas13 protein (dCas13) has been fused to ADAR2 to develop RNA base editors, such as REPAIR (RNA Editing for Programmable A to I Replacement)^224^ and RESCUE (RNA Editing for Specific C-to-U Exchange)^225^ that can be used to introduce A-to-I or C-to-U RNA editing, respectively. To minimize the substantial off-target RNA editing of REPAIRv1, the Zhang laboratory identified a new variant dCas13b-ADAR2_DD_ (E488Q/T375G), termed as REPAIRv2, enabling precise, efficient, and highly specific RNA base editing^224^.

More recently, by recruiting endogenous ADARs with different types of engineered ADAR-recruiting RNAs (arRNAs), next generation of RNA base editors, that is RESTORE (recruiting endogenous ADAR to specific transcripts for oligonucleotide-mediated RNA editing)^21^ and LEAPER (leveraging endogenous ADAR for programmable editing of RNA)^22^, have been developed to circumvent problems caused by ectopic expression of editing enzymes or ADARs protein. To improve the editing efficiency and reduce bystander off-target editing, CLUSTER^226^, cadRNAs^227^ and LEAPER 2.0^228^ were generated as the most advanced RNA base editors by using in silico-optimized CLUSTER guide RNAs^226^ or covalently closed circular arRNAs^227,228^, as well as by using CMV promoter instead of U6 promotor and the depletion of pairings of uridines with off-target adenosines^228^.

## Therapeutic and clinical considerations

### Considerations on therapeutic gene editing in patients

In this section we review the factors that are critical for the use of gene editing techniques to treat human diseases, including therapeutic benefits, efficiency and delivery in vivo, precision and specificity.

With regard to benefits, in general these could be considerable for gene therapies that rectify the underlying molecular lesion for genetic disease or target fundamental pathophysiologic features of acquired disease. The timing and therapeutic fraction of target cells to reverse the pathophysiology may vary depending on disease. For disease settings in which irreversible organ damage accumulates with time, providing gene therapy early in the disease course may be advantageous^229^. The therapeutic fraction may be estimated from clinical observations for diseases in which somatic mosaicism or chimeric transplantation outcomes have been described^230,231^; in other cases, this threshold may only emerge after therapeutic gene editing studies have been conducted. In addition, targeting a sub-threshold fraction of cells may provide an intermediate clinical benefit and some genetic modifications may partially ameliorate a disease, such as by converting a null to a hypomorphic allele^232^.

For efficient editing, adequate delivery systems are needed to achieve enough gene modifications (either monoallelic or biallelic depending on the disease setting) in the required fraction of cells. The efficiency depends not only on the intrinsic activity of the gene editor but also on its delivery method to cells. For ex vivo therapies - such as targeting hematopoietic cells - often the delivery is by pulse of RNP or mRNA/gRNA via electroporation^120^. For in vivo therapies, the most common delivery is by AAV^233^, although nanoparticles^234^ and bare RNPs^1^ may also be applied. Given that pulse delivery of gene editors to primary cells may result in a modest and short exposure, possible genomic effects as observed in vitro may overestimate possible editing effects. Some genomic repair unintended effects, like micronuclei formation or loss of heterozygosity^82,235^, are restricted based on cell cycle stage, so targeting non-dividing cells could possibly avoid these effects (as compared to targeting dividing cells during in vitro culture). Other genomic effects may depend on the cell state; for example, the frequency of retrotransposition effects may be related to the underlying cellular activity of retrotransposons^57^. Of note, therapies that target mRNA rather than DNA may require durable delivery or repeated administration.

Related to efficiency is precision, which enumerates how many modifications are perfect and how many are undesirable. For intended HDR repair, there might be concurrent NHEJ repair which will be produced in a fraction of cells. For example, accompanying HDR repair of the sickle cell disease mutation would be imperfect repair that might convert a sickle cell disease allele to a β-thalassemia allele, which could give a mixed clinical picture of chimeric healthy, sickle cell disease, and β-thalassemia HSCs each producing blood cells^236^. For NHEJ repair targeting gene regulatory elements, long deletions could remove coding sequences and lead to loss-of-function alleles^74^; for other applications in-frame indels could give neomorphic effects. Likewise for base editing, bystander edits or unintended substitutions (like C-to-G for a C-to-T base editor) could produce additional phenotypes, such as due to unintended missense or nonsense alleles in addition to therapeutic modifications.

Specificity describes the capacity for editors to produce changes elsewhere in the genome besides the intended target. Some of these off-target effects may be predictable and testable by different methods as described above. However, other effects may be unexpected, stochastic or widely distributed and thus could escape attention of targeted assays. Even whole-genome sequencing may be an insensitive assay given that in theory rare clonal events could be clinically meaningful. As the frequency of these events diminishes (ultimately to a single event in many targeted cells), it may become infeasible to detect them without targeted assays. Often there may be a tradeoff between gene editing efficiency, precision and specificity^237^. Thus, a short pulse of high-level expression may give a desirable balance of efficiency and specificity^121^.

Finally, one more challenge for the field is to identify the models to measure efficiency, precision and specificity. It can be difficult to comprehensively test genomic modifications in therapeutically relevant contexts with experimental models because it is expected that non-human cells would possibly have different off-target sequences compared to human cells given the different genomic sequence. Thus, experiments in non-human models may not fully mimic the in vivo conditions relevant to therapeutic delivery. Improved models, such as humanized mice, may allow testing on-target editing efficiency and off-target effects in certain tissues (like hematopoietic cells) but not in others. Thus, close follow-up and correlative molecular analyses from subjects enrolled in clinical trials may be the only way to truly evaluate comprehensive genomic editing outcomes. These analyses may be learned from studies focused on easily accessible tissues (like hematopoietic cells), from precious tissue biopsies when feasible to collect, and possibly from cell-free DNA^238^, but might be not feasible for other non-accessible tissues.

### Considerations on clinical risks of gene editing

Since gene editing approaches are still in an early phase of clinical development and no CRISPR-based therapy has been approved for use in patients yet^239^, their safety and associated clinical risks are uncertain. Although an increasing bevy of techniques may be employed to catalog on- and off-target genomic effects, a somatic genetic modification on its own may not portend clinical consequences. Cells spontaneously accumulate DNA damage over time and with cell divisions, so a modest one-time increase in acquisition of somatic genetic variation may not be particularly notable. Overall, most genetic variants are expected to be neutral with few variants that may be deleterious to cells being clinically inconsequential. In contrast, the potentially most concerning category would be those variants that cause a gain-of-function that could promote clonal expansion and tumorigenicity, via activating oncogenes or disrupting tumor suppressors. So far, no examples of unintended tumorigenicity of therapeutic gene editing have been reported to our knowledge in clinical studies, suggesting the frequency of these effects may be extremely low.

Finally, the clinical considerations must extend to the entirety of the therapeutic gene editing procedure, including any collateral risk of cellular delivery and host responses which may represent more likely classes of clinical risk. For in vivo approaches this could include unwanted host responses to gene transfer vectors like viruses (like AAV, AdV, LV, etc.) or transposons (Sleeping Beauty) that may cause stress responses, such as interferon responses and DNA damage responses^240,241^. In addition, it is known that therapies that require preparative conditioning, like the genotoxic alkylating myeloablative chemotherapy that often accompanies hematopoietic stem cell transplant or the lymphodepletion that attends immunotherapies, may have risks that need to be considered in the overall risk profile but are not attributable to gene editing per se. For example, two cases of AML were observed out of 47 patients undergoing a gene therapy approach for sickle cell disease^242,243^. Molecular analyses suggest that insertional mutagenesis was not the cause for these malignant events, so narrowly one could conclude that the gene therapy did not cause the malignancies. In fact, lentiviral therapies for monogenic blood disorders have a highly favorable safety profile without genotoxicity events observed over greater than 700 patient-years, despite their obligate delivery of semi-random genomic insertions to target stem cells^244^. This observation illustrates that tallying genomic modifications does not easily translate into quantifying clinical risk.

## Conclusions and future perspectives

The past 10 years have witnessed remarkable progress in understanding and manipulation of CRISPR-Cas systems, especially in the field of genome editing and RNA editing. CRISPR–Cas9/Cas12/Cas13 nucleases, DNA base editors, prime editors, and RNA base editors have revolutionized the life sciences, paving the way for advances in basic research and therapeutic applications. Nevertheless, future work is needed to provide insights into more precise, more efficient and safer editing tools. Besides DNA and RNA editing, other applications of CRISPR-based tools include epigenome and gene regulation, functional screening, and diagnostics have been well-summarized in recent reviews^245,246^.

There are plenty of sensitive and unbiased methods that have been developed to evaluate the off-target concerns during genome editing, pushing the limit of editing specificity and efficiency. Despite progress, the currently available methods to detect off-target activity of CRISPR-Cas systems are still heterogenous, not extensively compared one to another, and optimized only for few experimental systems. Furthermore, a method validated and approved for clinical use is lacking. Therefore, the challenge is to develop a consensus method that can unbiasedly, sensitively, rapidly and cost-effectively detect the CRISPR-Cas off-targets in most cellular system, including in vivo models. This need will grow even further when the therapeutic applications of CRISPR-Cas will become of broader use. Improved methods to detect off-target activity will guide the efforts for the development of high-fidelity Cas nuclease or cytidine/adenine deaminases with minimized Cas-dependent or Cas-independent off-target editing, shedding light on the future direction of precision medicine. Of note, these minimized off-target effects focus on not only DNA level but also the RNA level, especially for the DNA base editors that may cause substantial RNA off-targeting.

Unlike DNA editing, RNA editing offers an alternative to genome editing in certain therapeutic applications in a reversible and tunable manner, especially for the RNA base editors that do not need to introduce foreign Cas nuclease or exogenous ADARs protein into edited cells. Over-expression of Cas or ADARs proteins is associated with several safety concerns, including substantial genome-wide and/or transcriptome-wide off-target, immunogenicity, oncogenicity, and delivery hurdles^228^. Nonetheless, future work on specificity and efficiency are still needed to improve the RNA base editing, as well as more advanced RNA base editors or “RNA prime editors” that could modify the RNA in a broader way to further illuminate RNA biology. In addition, other types of RNA editing tools are still need to be developed for specific and safe RNA editing applications, like recently reported RESTART^247^ which relies on guide snoRNA to revert PTC-induced translation termination. On the other hand, “endogenous DNA base or prime editors” that without the need of foreign Cas nucleases or exogenous deaminase proteins would be an exciting breakthrough for next-generation genome editing.

Finally, for any of these considerations to be relevant a therapy must be available to patients. Given that many patients with the genetic disease may have rare or ultra-rare mutations, it may be extremely challenging to develop a personalized therapy. The current regulatory path raises a substantial barrier in terms of the requirement for extensive pre-clinical studies and high cost of goods, which may be of uncertain prognostic importance. Perhaps new regulatory paths could catalyze the development of additional gene therapies, in particular for rare indications, that could maintain a focus on safety while enabling patient access to innovative and complex therapies. Once a therapy enters clinical trials, typically patients must meet strict inclusion/exclusion criteria, so the applicability of novel therapies to different types of patients, including those with more severe disease, may be difficult to extrapolate. After a therapy is approved, the high cost and complexity of therapies may represent a challenge to patient access and scalability. All things being equal, cheaper and simpler therapies would be easier for patients to access.
